# One-Shot Multi-Path Planning Using Fully Convolutional Networks in a Comparison to Other Algorithms

**DOI:** 10.3389/fnbot.2020.600984

**Published:** 2021-01-08

**Authors:** Tomas Kulvicius, Sebastian Herzog, Timo Lüddecke, Minija Tamosiunaite, Florentin Wörgötter

**Affiliations:** ^1^Third Institute of Physics - Biophysics, Department for Computational Neuroscience, University of Göttingen, Göttingen, Germany; ^2^Faculty of Computer Science, Vytautas Mangnus University, Kaunas, Lithuania

**Keywords:** multi-source single-target path planning, multi-agent systems, robotics, neural path planning, mazes

## Abstract

Path planning plays a crucial role in many applications in robotics for example for planning an arm movement or for navigation. Most of the existing approaches to solve this problem are iterative, where a path is generated by prediction of the next state from the current state. Moreover, in case of multi-agent systems, paths are usually planned for each agent separately (decentralized approach). In case of centralized approaches, paths are computed for each agent simultaneously by solving a complex optimization problem, which does not scale well when the number of agents increases. In contrast to this, we propose a novel method, using a homogeneous, convolutional neural network, which allows generation of complete paths, even for more than one agent, in one-shot, i.e., with a single prediction step. First we consider single path planning in 2D and 3D mazes. Here, we show that our method is able to successfully generate optimal or close to optimal (in most of the cases <10% longer) paths in more than 99.5% of the cases. Next we analyze multi-paths either from a single source to multiple end-points or vice versa. Although the model has never been trained on multiple paths, it is also able to generate optimal or near-optimal (<22% longer) paths in 96.4 and 83.9% of the cases when generating two and three paths, respectively. Performance is then also compared to several state of the art algorithms.

## 1. Introduction

Path planning is defined as the problem of finding a temporal sequence of valid states from an initial to a final state given some constraints (Latombe, 2012). For autonomous vehicles, this corresponds to a path finding problem from the vehicles' current location to a destination, while avoiding obstacles or other agents. For robotic manipulation, one needs to plan a motion trajectory to perform collision free motion. Thus, trajectory- or path-planning is a fundamental issue in a wide variety of applications. In this work, we specifically address the issue of multi-path planning for single agents as well as single path planning for multi-agent systems.

Most common classical approaches for path planning are the Dijkstra algorithm (Dijkstra, 1959), the A^*^ search (Hart et al., 1968), and its variants (e.g., see Korf, 1985; Koenig et al., 2004; Sun et al., 2008; Harabor and Grastien, 2011). Dijkstra and A^*^ algorithms perform well on grid-based representations and provide the optimal solution (i.e., shortest path). However, they do not scale well with increased dimensions and path lengths.

Another class of common approaches, are sampling based methods such as the rapidly-exploring random tree algorithm (RRT, LaValle, 1998) and it is variants (e.g., see Karaman and Frazzoli, 2011; Islam et al., 2012; Gammell et al., 2015, 2020). While RRTs are more suitable for sparse continuous spaces, they do not perform so well on grids or in complex environments like mazes as compared to Dijkstra or A^*^ (Knispel and Matousek, 2013; Bency et al., 2019), i.e., paths are not necessarily optimal. Also these methods are computationally more expensive in maze-like environments and require parameter tuning to obtain optimal performance, whereas Dijsktra and A^*^ are parameter-free methods. Some approaches also exist that employ deep learning methods to learn heuristic search or to select an algorithm for a heuristic search in order to minimize search effort (Bhardwaj et al., 2017; Sigurdson and Bulitko, 2017).

Some other path planning approaches are based on bio-inspired neural networks (Glasius et al., 1995, 1996; Yang and Meng, 2001; Bin et al., 2004; Li et al., 2009; Qu et al., 2009; Chen and Chiu, 2015; Rueckert et al., 2016; Ni et al., 2017). Neurons in these networks represent specific locations in the environment similar to place cells found in hippocampus (O'Keefe and Dostrovsky, 1971). The optimal path is found by activating the target neuron and propagating activity to the neighboring cells until the source neuron is reached. The path is reconstructed by following the activity gradient of the network from the source to the target. Conceptually, these methods are similar to the Dijkstra algorithm and will find the optimal solution, however, they require several iterations until convergence.

In case of path planning for multi-agent systems, usually, decentralized approaches are used (e.g., see Wang and Botea, 2011; Desaraju and How, 2012; Chen et al., 2017; Long et al., 2017; Everett et al., 2018), where path search is performed for each agent separately. Thus, computation time scales with the number of the searched paths (i.e., the number of agents).

Recently, several path planning methods have been proposed using shallow (De Momi et al., 2016) or deep network approaches such as deep multi-layer perceptrons (DMLP; Qureshi et al., 2019), long short-term memory (LSTM) networks (Bency et al., 2019), and deep reinforcement learning (deep-RL; Tai et al., 2017; Panov et al., 2018), or mixed approaches that can be used for robot movement generation (Seker et al., 2019). All these methods plan paths iteratively by predicting the next state or next action (in case of RL) based on the current state, environment configuration, and the target position until the goal is reached. Therefore, the network has to be exploited many times until a complete path can be constructed.

Humans, however, can directly see a path in simple, quite empty mazes without having to perform serial search. Everyone knows this from own experience and, commonly, such phenomena are subsumed under the term *perceptual pop-out*. Serial search sets in as soon as mazes get more complex (Crowe et al., 2000; Chafee et al., 2002) and this is evidenced, for example, by saccades that do not exist during pop-out but occur during serial search (Chafee et al., 2002). Thus, in the current study we asked, weather it would be possible to implement path pop-out also in an artificial, network-based system. From the above discussed history of the path-finding literature, it appears unorthodox to think so, but: Can one use pure filtering processes to achieve something similar to a path pop-out?

To show this, here we present a method based on a fully convolutional network, coined CNPP—Convolutional Network for Path Planing, which allows multi-path planning in one-shot, i.e., multiple path search^1^ can be realized with a single prediction iteration of the network which allows performing path search faster compared to other approaches. Note that, although the model was not trained on multiple paths (only single paths were used for training), CNPP is able to generate optimal or near-optimal paths in 96.4 and 83.9% of the cases when generating two and three paths, respectively.

The structure of this manuscript is as follows. In the following section we explain our approach (section 2), then we provide an evaluation of our method where we first analyze the general performance of the proposed network and afterwards we analyze path planning of multiple paths in simulated environments. This is done by comparing it to four other powerful algorithms for path planning. Finally, we provide an application of our approach for navigation in real cities (section 3). In section 4, we relate our approach to the comparison methods and especially discuss also under which conditions the different, here-investigated algorithms might be beneficial and under which conditions they ill-perform. This way we hope to provide useful insights to scientists who wish to apply the different methods^2^.

## 2. Proposed Method

Our approach works in 2D and in 3D. In the following, the formalism is defined for the 2D case, but it applies in a similar way to 3D, too.

### 2.1. Overview

The task is to predict an optimal path (or several paths) in a so far unseen, new environment given any start- and goal-point. The new environment can be of different size and/or can consist of different configurations and/or shapes of obstacles as compared to the ones used in the training set. In this study, we have considered both 2D and 3D environments.

**Figure 1:**
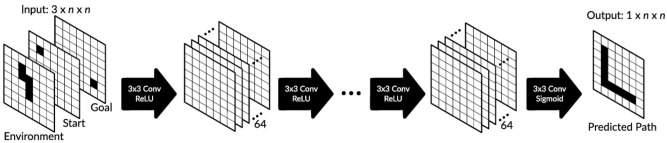
Proposed network architecture based on a fully convolutional neural network. If not otherwise mentioned, we used 21 convolutional layers with 3 × 3 filters (64 filters in layers 1–20 and one filter in the last layer). We used ReLU activation units in all but the last layer where sigmoid activation units were used.

Each environment is described by an occupancy grid (binary image) of size *n* × *m* in 2D, where we mostly use *m* = *n* (similar in 3D), where free spaces are marked in white and not traversable spaces (obstacles) are marked in black. The start- and end-points are represented by separate binary images where the start- (or end-point) is marked black and all other grid cells are marked white (see input in Figure 1).

After training, the network is able to predict a collision-free path from the start- to the end-point, which is represented as a binary 2D or 3D image where the path is marked again in black (see output in Figure 1 for the 2D example). The actual trajectory, i.e., the sequence of locations that the agent needs to traverse, is then constructed by tracking the black cells using forward- (from the start-point) and backward- (from the end-point) search until the path-segments meet or cross each other.

In general, it would possible to employ a fully convolutional network in a fully end-to-end manner on raw images but the network would need many more layers and a direct comparison to other state-of-the-art planning algorithms such as A^*^, RRT, BIT, which do not work on raw images, would not be possible. To create a network that operates on raw images would essentially amount to a mixed approach that performs image processing and path finding, which is not the purpose of this study. Instead, here we target path planning for multi-agent systems where the map is known but positions of sources and targets change over time.

Typical 2D path planning applications offer map information directly. These are, for example, taxi-scenarios, such as that shown in Figure 8, navigation in indoor environments, e.g., shopping malls, path planning for multi-players in games. Here, usually maps are available (e.g. city maps, floor maps, etc.) and can be binarized just by using simple color thresholding, where—by contrast—using any network-based approach to do this seems too effortful.

In case of 3D, we aim at trajectory planning for robotic manipulators. In these cases, depth sensors are usually used, which generate 3D point cloud data, from which the generation of binary 3D images is also straightforward, i.e., 3D points in the point cloud data would correspond to obstacles and empty spaces would correspond to free spaces.

### 2.2. Data

#### 2.2.1. Input Definition

We define the grid map as a binary image *I*^*e*^, where we set $I_{i,j}^{e}=0$, if the grid cell (*i, j*) is free (no obstacles), otherwise we set $I_{i,j}^{e}=1$, if it contains obstacles. Similarly, we also define maps for start- and end-points: we set $I_{i,j}^{s/g}=1$ at the start-/end-point and we set $I_{i,j}^{s/g}=0$ anywhere else. Here *I*^*s*^ and *I*^*g*^ denote the start map and the goal map, respectively. Thus, we obtain an input of size 3 × *n* × *m*.

We have also analyzed the network's capability to plan multiple-paths at once from several start-positions to the same goal position. In these cases several grid cells of $I_{i,j}^{s}$ were set to 1 to mark start-positions of the agents.

#### 2.2.2. Output Definition

As in case of the input maps, we used a binary map to define the output map *O*, where we set *O*_*i,j*_ = 1, if the found path was traversing this grid cell (*i, j*), and we set *O*_*i,j*_ = 0 everywhere else. In our study, we used the A^*^ grid search algorithm to find the optimal solutions (ground truth paths for training), where eight movement directions (vertical, horizontal, and diagonal) were allowed. To calculate the cost of the path from the start to the current grid cell, for vertical and horizontal moves we used a cost of 1 and for diagonal moves we had cost of $\sqrt{2}$. We used the Euclidean distance from the current grid cell to the goal cell to calculate the cost of the path from the current grid cell to the goal.

#### 2.2.3. Data Generation

The input maps for training and testing were generated randomly in the following way. In case of 2D grids, each grid cell was set to 1 (an obstacle) with a probability *p*_*o*_ = 0.6 or was set to 0 (a free space) with a probability *p*_*f*_ = 1 − *p*_*o*_. Note that two diagonal configurations of obstacles were not permitted: $\left\{I_{i,j}^{e}=0,I_{i,j+1}^{e}=1,I_{i+1,j}^{e}=1,I_{i+1,j+1}^{e}=0\right\}$ and $\left\{I_{i,j}^{e}=1,I_{i,j+1}^{e}=0,I_{i+1,j}^{e}=0,I_{i+1,j+1}^{e}=1\right\}$, as this would have allowed the A^*^ algorithm to generate paths going between two diagonally arranged obstacles that touch each other only by a corner, which in real scenarios, however, cannot be traversed. As a consequence, environments mostly consist of vertical or horizontal bar-like structures. In case of 3D grids, we assumed that objects (obstacles) do not float in the air. Thus, obstacles were columns of random height *h* (from a uniform distribution), i.e., $I_{i,j,1\ldots h}^{e}=1$.

We generated three data sets: two data sets (2D grids and 3D grids) for learning and testing predictions of single paths and one data set for testing predictions of multiple paths (up to three start-positions). The procedure for generating training data and test data was the same as explained above, however, we performed a sanity check to make sure that none of the maps from test set are identical with the maps in the training set. Also, note that we did not train our network on multiple paths.

**Figure 2:**
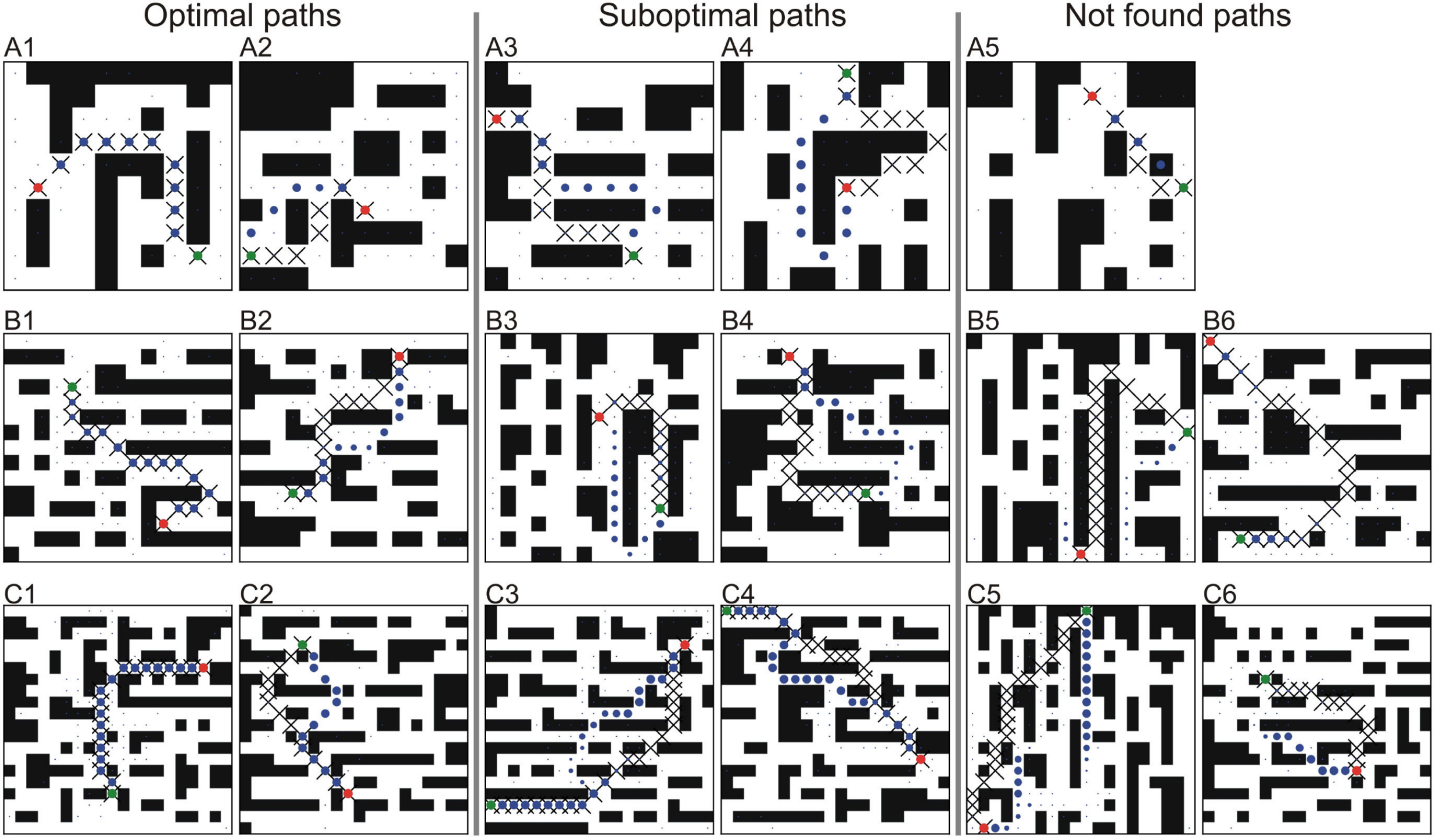
Examples of single path predictions on unseen environments for different square grids trained and tested on the same grid size. **(A)** *n* = 10, **(B)** *n* = 15, and **(C)** *n* = 20. The first two columns show optimal (shortest) paths, the middle two columns sub-optimal paths and the last two columns not found paths. Crosses denote the A^*^ solution, where blue dots denote the predicted path using our CNPP. Size of the dots corresponds to small (close to zero) and large values (close to one) of the network outputs. Green and red dots correspond to the start- and end-point, respectively.

For the first two cases, we generated environments of three different sizes: 10 × 10, 15 × 15, 20 × 20, and 30 × 30 (the latter only for the 2D case). Some examples of 15 × 15 environments are shown in Figure 2. We generated 30,000 environments for each 2D case and 97,000 for each 3D case with different obstacle configurations and different random start- and end-points. Note that the minimum Euclidean distance between start- and end-points was 5 to avoid very short paths and to exclude trivial cases.

For the prediction of multiple paths, we generated 1,000 environments of size 15 × 15 with different obstacle configurations but fixed start- and end-positions. Start-points were located at positions (1, 1), (1, *n*), and (*n*, 1) (in the corners) where the goal was at the position (8, 8) (in the middle). We have chosen such configuration on purpose in order to keep all sources as much apart as possible and as far from the target as possible. Some examples of environments are shown in Figure 6.

### 2.3. Network

#### 2.3.1. Network Architectures

For the 2D case, we used a fully convolutional network as shown in Figure 1. The input layer consists of three 2D binary images and the output is a single 2D binary image as described above. We used 20 identical 2D convolutional layers with 64 filters of size 3 × 3 and with stride 1 and one convolutional layer at the end with one filter of size 3 × 3. The first layer maps input of size 3 × *n* × *n* to 64 feature maps of size *n* × *n* where the same 2D filters of size 3 × 3 are used for all three input layers. After each convolutional layer we used a batch normalization layer (not shown), and after the last convolutional layer we added a dropout (10%) layer. Note that dropout was only used in training. In all but the last convolutional layer we used ReLU activation units, whereas in the last convolutional layer sigmoid activation was used. We define the network output as Ô, where ${\hat{O}}_{i,j}$ can obtain values between 0 and 1. In all layers, we used zero padding to keep the same dimension and prevent information loss. Note that we also tried a network architecture with non-zero padding, but performance was worse (results not shown).

For the 3D case, we used a similar network architecture with 3D convolutional layers (filters of size 3 × 3 × 3), except that we used more filters in the first four layers (1,024, 512, 256, and 128) to cover larger variability of possible filters due to the additional dimension.

#### 2.3.2. Training Procedure

We used the mean squared error (MSE) between the network output Ô and the ground truth solution *O* as loss. The ADAM optimizer with default learning parameters was used for training both 2D and 3D networks. For the 2D case we used a batch size of 64 samples and for the 3D case (due to our hardware limitations) we used a batch size of 16 samples. Early validation stopping was used if the accuracy on the validation set did not increase within the last 10 epochs to prevent over-fitting. We used 28,000 environments (26,000 for training and 2,000 for validation) and 95,000 environments (90,000 for training and 5,000 for validation) for training the 2D network and 3D network, respectively. We used 2,000 unseen environments for testing the performance of both networks. We trained the 2D network ten times and then selected the best model with the highest accuracy on the validation set. Since we did not observe high variation of MSE when training the 2D network, we trained the 3D network only once.

### 2.4. Path Reconstruction

As explained above, the output of the network is a value map (grid), which contains values between zero and one. These values can be treated as certainty of the grid-cell to be part of the path. The output map as such does not yet provide the temporal sequence of points that would lead from the start-point to the end-point (goal). Thus, path(s) has/have to be reconstructed from this prediction-map. In our case, we simply did this by using bidirectional search: Let us denote the forward and the backward part of each path *k*(*k* = 1…*K*) as a temporal sequence of points on the 2D grid with $\left(x_{t}^{f,k},y_{t}^{f,k}\right)$ and $\left(x_{t}^{b,k},y_{t}^{b,k}\right)$, respectively. We also annotate the start-point of each path as $\left(x_{s}^{k},y_{s}^{k}\right)$ and the end-point (goal) as (*x*_*g*_, *y*_*g*_). Initially we set $\left(x_{1}^{f,k},y_{1}^{f,k}\right)=\left(x_{s}^{k},y_{s}^{k}\right)$ and $\left(x_{1}^{b,k},y_{1}^{b,k}\right)=\left(x_{g},y_{g}\right)$. Given the current position $\left(x_{t}^{k},y_{t}^{k}\right)$ of the forward/backward path *k*, the next position of the forward/backward path *k* is obtained by choosing the grid cell (*i, j*) in the nearest neighborhood of the current position (we used eight nearest neighbors) with maximum value of the network output Ô:

(1) $$\begin{array}{l} \left(x_{t+1}^{k},y_{t+1}^{k}\right)=\arg\underset{i,j}{\max}{\hat{O}}_{i,j}, \end{array}$$

where $\left\{i\neq x_{t}^{k},j\neq y_{t}^{k},i\in \left\{x_{t}^{k}-1,x_{t}^{k}+1\right\},j\in \left\{y_{t}^{k}-1,y_{t}^{k}+1\right\}\right\}$. After this step, we set Ô_*i,j*_ = 0. We continue constructing forward and backward paths until one of the three conditions is met:

1. End-point is reached, $\left(x_{t}^{f,k},y_{t}^{f,k}\right)=\left(x_{g},y_{g}\right)$;
2. Start-point is reached, $\left(x_{t}^{b,k},y_{t}^{b,k}\right)=\left(x_{s}^{k},y_{s}^{k}\right)$;
3. The paths cross each other, $\left(x_{t}^{f,k},y_{t}^{f,k}\right)=\left(x_{h}^{b,k},y_{h}^{b,k}\right)$ or $\left(x_{t}^{b,k},y_{t}^{b,k}\right)=\left(x_{h}^{f,k},y_{h}^{f,k}\right\}$, where 1 ≤ *h* ≤ *t*.

Thus, depending on the condition met, the final path *P*^*k*^ is constructed as follows:

(2) $$\begin{array}{l} P^{k}=\left[\left(x_{1}^{f,k},y_{1}^{f,k}\right),\ldots ,\left(x_{N_{f}}^{f,k},y_{N_{f}}^{f,k}\right)\right],\text{if condition 1. is met}; \end{array}$$

(3) $$\begin{array}{l} P^{k}=\left[\left(x_{N_{b}}^{b,k},y_{N_{b}}^{b,k}\right),\ldots ,\left(x_{1}^{b,k},y_{1}^{b,k}\right)\right],\text{if condition 2. is met}; \end{array}$$

(4) $$\begin{array}{r} P^{k}=\left[\left(x_{1}^{f,k},y_{1}^{f,k}\right),\ldots ,\left(x_{t}^{f,k},y_{t}^{f,k}\right),\left(x_{h-1}^{b,k},y_{h-1}^{b,k}\right),\ldots ,\left(x_{1}^{b,k},y_{1}^{b,k}\right)\right]\text{or}\\ \left[\left(x_{1}^{f,k},y_{1}^{f,k}\right),\ldots ,\left(x_{h-1}^{f,k},y_{h-1}^{f,k}\right),\left(x_{t}^{b,k},y_{t}^{b,k}\right),\ldots ,\left(x_{1}^{b,k},y_{1}^{b,k}\right)\right],\\ \text{if condition 3. is met}. \end{array}$$

Here, *N*_*f*_ and *N*_*b*_ are the lengths of the forward and the backward path, respectively. In real applications, for example in robotics, the points of the path *P* could then be used as via points to generate trajectories using conventional methods such as splines (Egerstedt and Martin, 2001; Siciliano et al., 2009) or more advanced state-of-the-art methods such as dynamic movement primitives (DMPs, Ijspeert et al., 2013). Note that in some cases paths could not be reconstructed, i.e., none of the stopping conditions were met. In this case we treated network's prediction as “path not found.”

## 3. Experiments

We compared our approach against four algorithms: the A^*^ algorithm (Hart et al., 1968), the RRT algorithm (LaValle, 1998), and Deep Multi Layer Perceptron (DMLP; Qureshi et al., 2019) and the BIT^*^ (Batch Informed Trees; Gammell et al., 2020). For our network implementation we used Tensorflow and Keras API^3^, where for DMLP we used PyTorch implementation provided by the authors. A^*^ and RRT algorithms were implemented in Python. For evaluation we used a PC with Intel Xeon Silver 4114 (2.2 GHz) CPU and NVIDIA GTX 1080 (11 GB) GPU.

### 3.1. Evaluation Measures

For comparison we used the following criteria: (1) success rate, (2) path optimality, and (3) run-time of the algorithm. All measures have been applied to our method (CNPP) but also to all other algorithms used for comparison.

#### 3.1.1. Success Rate

We counted an algorithm's prediction as successful (path found) if the path *P* could be reconstructed from its output, otherwise we counted the prediction as failed (path not found). The success rate *SR* was computed as percentage of successfully found paths out of all tested environments:

(5) $$\begin{array}{l} SR=100\%\cdot \frac{N_{S}}{N_{T}}, \end{array}$$

where *N*_*S*_ is the number of successfully found paths and the *N*_*T*_ is the total number of path planning queries.

#### 3.1.2. Path Optimality

We also checked whether successfully predicted paths were optimal (i.e., shortest path) or not. For that, we compared path lengths of the paths obtained by using the different algorithms, where A^*^ renders always an optimal path on the octile grid and is used as the ground truth. The path length was computed as follows:

(6) $$L=\sum_{t=1}^{q-1}||P_{t+1}-P_{t})||,$$

where *P*_*t*_ = (*x*_*t*_, *y*_*t*_) are the points of the reconstructed path from network's output Ô, and *q* is the number of points in the path sequence. Here, ||·|| is the Euclidean norm. The path predicted by the any algorithm (sub-script: …_*alg*_) was counted as optimal if *L*_*alg*_ ≤ *L*_A*_. Note that RRT based algorithms can produce shorter paths than A^*^ since they operate in continuous space. Consequently, the percentage of optimal paths *OP* was computed as:

(7) $$\begin{array}{l} OP=100\%\cdot \frac{N_{O}}{N_{T}}, \end{array}$$

where *N*_*O*_ is the number of optimal paths. The paths predicted by an algorithm are not always optimal, thus, we also computed the path length ratio *LR* of non-optimal paths to analyze how much longer non-optimal paths are, compared to the A^*^ solutions:

(8) $$\begin{array}{l} LR=\frac{L_{alg}}{L_{A^{*}}}. \end{array}$$

#### 3.1.3. Algorithm Run-Time

For run-time comparison we analyzed how much time it takes to find a single path for different path lengths. In this evaluation, the path length was measured in steps, i.e., how many steps it takes to move from the start-point to the end-point.

### 3.2. Evaluation Procedures

We performed two types of experiments: (1) prediction of single paths (one source and one target) in 2D and 3D environments and (2) prediction of multiple paths (up to three sources and one target). In the first case, we were interested in the general performance of the system with respect to the above introduced measures and we wanted to check how well the network can generalize to different grid sizes. For this, we trained the network to predict single paths on three different grids (10 × 10, 15 × 15, and 20 × 20) and then tested each model on all three grids.

In the second type of experiments, we tested whether the network can predict multiple paths although the network has only been trained on single paths. In this study we checked the network's ability to predict up to three paths. Here we tested path predictions on a grid size 15 × 15, thus, the network model trained on 15 × 15 grid for single paths was used for testing.

### 3.3. Application to City Maps

Finally, we also applied and tested our proposed approach on real city maps. We assumed a “taxi scenario” where the task is to find a taxicab that is closest to a customer in terms of route distance in a given city scenario. For that we have chosen some parts of several city maps from the Moving AI Lab path finding database (Sturtevant, 2012). In this case, we predicted paths from four sources to one target, and then we found the closest source to the target based on the path length. As in the multi-path simulation experiments we placed sources in the corners and target in the middle. Such a configuration excluded an easy way of finding the closest source based on the direct Euclidean distances between sources and the target. The manually selected parts were down-sampled five times to 25 × 25 grids and tested on the 2D network that was trained on 20 × 20 grids.

## 4. Results

### 4.1. Parameter Analysis

Our approach uses only three hyper-parameters and, first, we provide an analysis of the network performance with respect to all of them: number of layers, kernel size, and number of kernels. In the first set of experiments we trained and tested three networks with different number of layers, i.e., 11, 21, and 31, on three grids of size 10 × 10, 20 × 20, and 30 × 30. Results are shown in Table 1 where we show average *SR* and *OP* obtained from 10 sample sets (200 samples per set).

**Table 1:** Analysis of network performance with respect to number of layers and kernel size, and number of kernels obtained from 10 sample sets (200 samples per set) on unseen 2D environments.

	**Number of layers**		
	**11**	**21**	**31**
**Success rate (%)**			
10×10	**100**	100	99.95
20×20	97.30	99.60*	99.80
30×30	94.10	96.20*	99.65*
**Optimal paths (%)**			
10×10	95.85	99.85*	97.05
20×20	59.04	86.55*	83.87
30×30	29.29	63.36*	79.43*
**Kernel size**			
**(20×20** **grid, 21 layers)**			
2×2	3×3	4×4	5×5
**Success rate (%)**			
70.10	99.60*	99.50	97.20
**Optimal paths (%)**			
8.13	86.55*	85.62	87.03
**Number of kernels**			
**(20×20** **grid, 21 layers)**			
16	32	64	96
**Success rate (%)**			
99.40	97.70	99.60*	97.85
**Optimal paths (%)**			
74.50	83.17*	86.55*	88.55

*The asterisks mark statistically significant increase of the values compared to the values shown in the previous column (i.e., on the left side; t-test, p <0.05). All other changes are not reaching significance. Bold numbers denote the highest significant improvement compared to the numbers shown in previous columns*.

When looking at the success rate (top part of the table), bold numbers show for which number of layers the success rate for the first time reached more than 99%. They follow the diagonal. This demonstrates that for larger grids deeper architectures are needed, where the number of layers should minimally correspond to the linear grid size. Architectures deeper than that (to the right from the diagonal) can sometimes improve performance (e.g., see grid 20 × 20, 31 layers), but only statistically insignificantly, and they will slow down calculations.

The suggestion to configure the network by following the diagonal essentially (except grid 10 × 10) also holds for the number of optimal paths.

In the second and the third set of experiments we trained and tested four networks (21 layers each) on a grid of size of 20 × 20 with different kernel sizes 2 × 2, 3 × 3, 4 × 4, and 5 × 5, and different number of kernels 16, 32, 64 and 96. Results demonstrate that the best performance is obtained with 64 kernels of size 3 × 3 when taking both success rate and percentage of optimal paths into account. Thus, in all further experiments networks with 64 (3 × 3) kernels were used.

Of course, it would also be possible to use architectures with different kernel sizes and different number of kernels for different layers. This creates a wide variety of different possible architectures, but given the high success rate found with the much simpler uniform architecture, we did not investigate such combinations.

### 4.2. Analysis of the Network's Internal Activity

To understand how the network finds a shortest path, we performed an additional analysis where we looked at the activity within feature maps for three different environments of size 20 × 20, i.e., without obstacle, with a rectangular obstacle and with an inverted u-shape obstacle. In all cases, a network with 21 layers trained on environments of size 20 × 20 was used as explained above.

**Figure 3:**
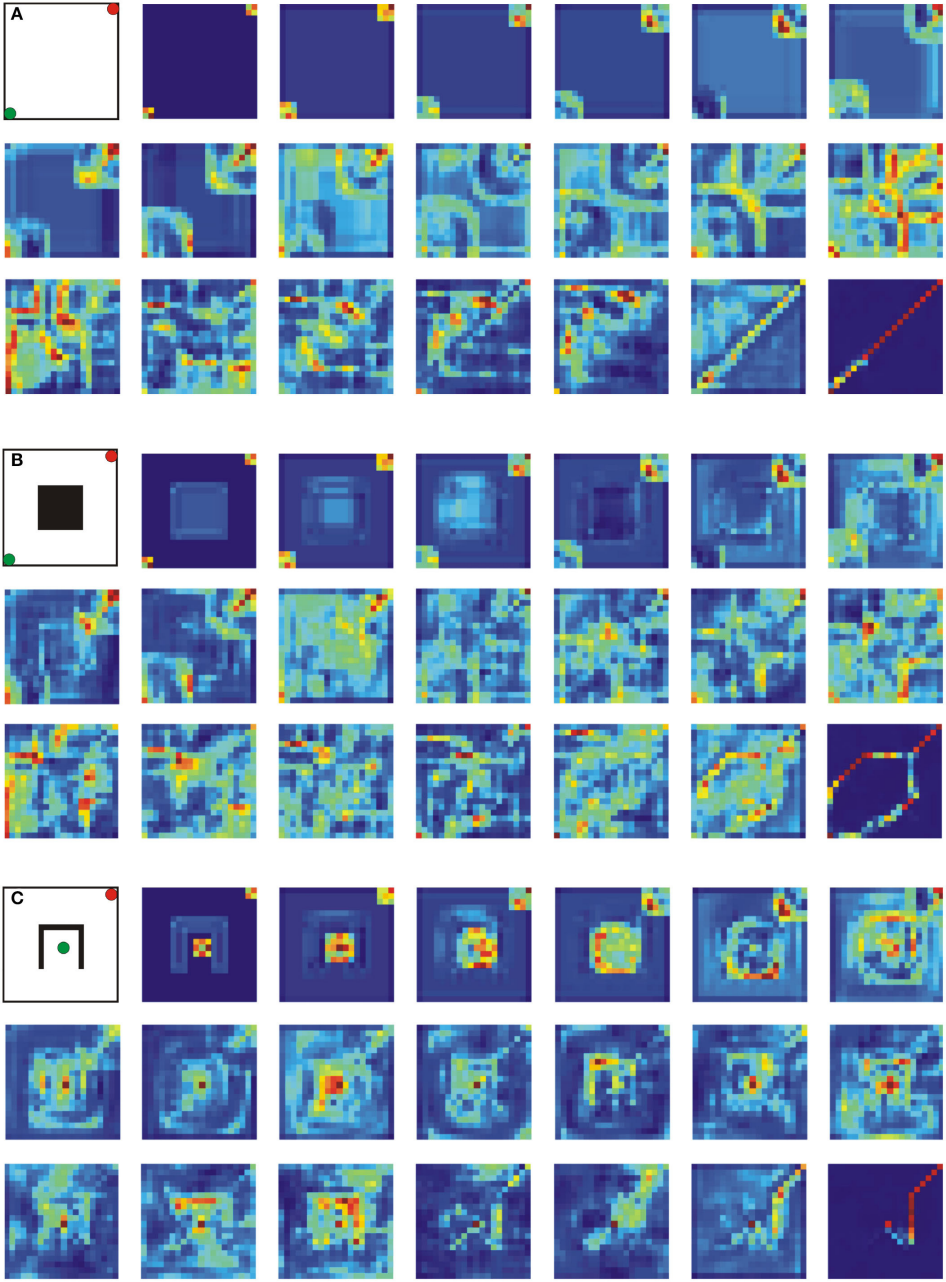
Analysis of the network's internal activity on three different environments of size 20 × 20. A network architecture with 21 layers was used in all three cases. **(A)** Environment without obstacle, **(B)** environment with a rectangular obstacle in the center, and **(C)** environment with an inverted u-shape obstacle in the center. Green and red dots correspond to start- and end-points, respectively. Each activity map shows the contribution of all 64 feature maps (sum over the feature maps) for layers 1–19, and 21. Blue and dark colors correspond to high and low activity, respectively.

Results are shown in Figure 3 where we plot the summed activity of all 64 feature maps (i.e., contribution of all filters) for layers 1–19 and 21. One can observe that overall activity is propagating outwards from the start and goal positions simultaneously in a wave-like manner (see case A), whereas other feature maps also respond to obstacles (compare first rows in cases A and B/C), which presumably blocks activity propagation at the obstacles and forces the activity to go around them (see second and third rows in cases B and C). Interestingly, such activity propagation is to some extent similar to the one observed in the wave-front expansion algorithm (Choset et al., 2005).

### 4.3. General Performance of the Proposed Network

Examples of single path predictions on different 2D grids are shown in Figure 2, where the network prediction is marked by blue dots^4^.

In most of the cases the network is able to predict optimal paths. Examples of optimal paths that corresponds to the A^*^ solution are shown in the first column, whereas in the second column we show examples of optimal paths that differ from the A^*^ path (usually going around an obstacle from the other side). In the third and fourth column we show feasible paths that are sub-optimal, i.e., longer (in most of the cases <10%) than those found by A^*^. The last two columns show cases of failed predictions, i.e., paths that could not be reconstructed from the network's output. This case only happened once for the 10 × 10 grid.

**Figure 4:**
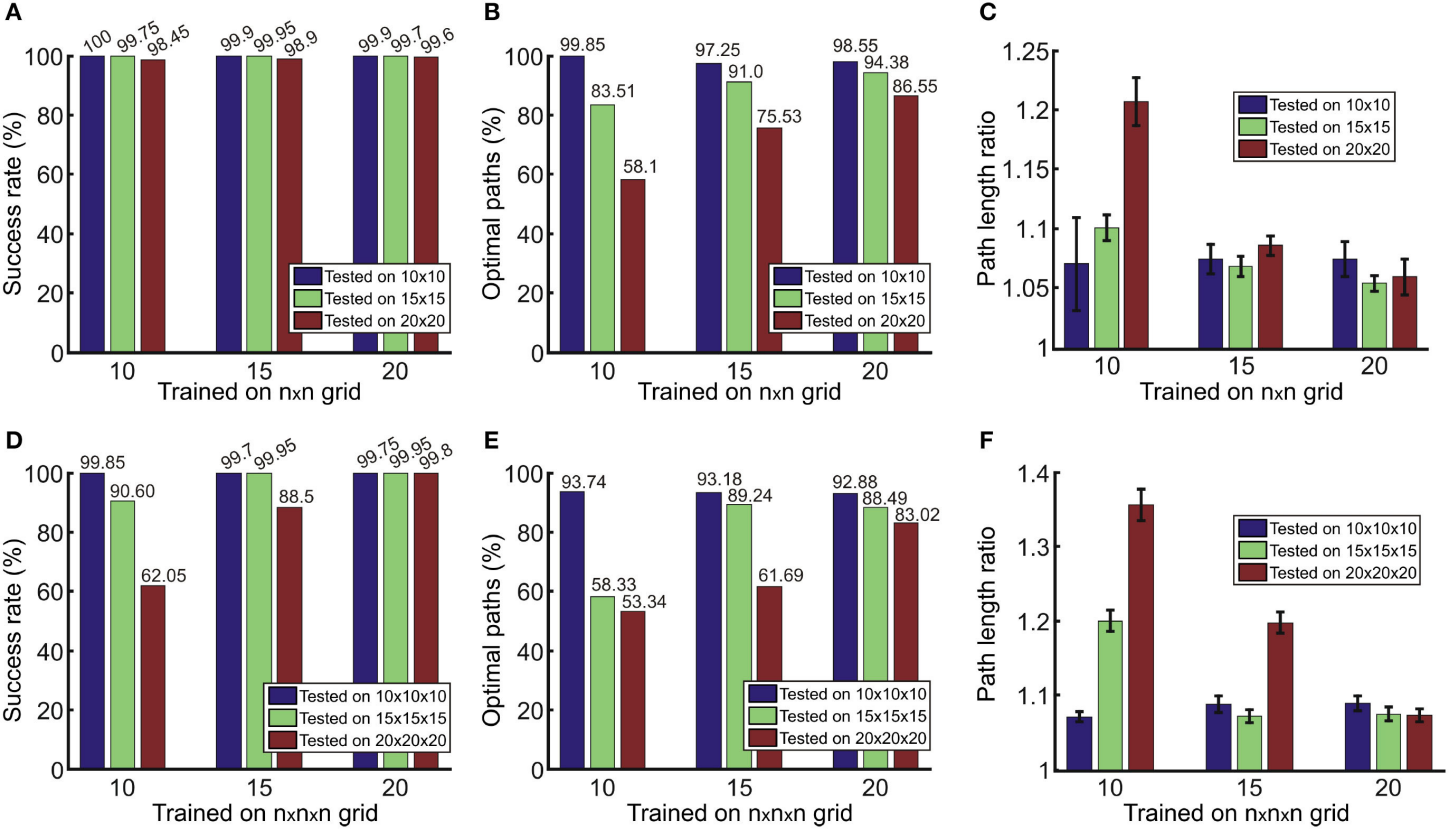
Results for the prediction of single paths in **(A–C)** 2D and **(D–F)** 3D environments obtained from 2,000 unseen environments for each case. Error bars in **(C,F)** denote confidence intervals of the mean (95%).

A statistical evaluation of single path predictions on simulated 2D and 3D environments is presented in Figure 4, where we show the networks' performances when trained and tested on different grid sizes. In general, we obtained a relatively high success rate (Figures 4A,D), above 99.5% for both, 2D and 3D, cases when trained and tested on the same grid size. Results demonstrate that the network can also predict paths quite reliably on a grid size that was not used for training. Except for cases trained on the grid sizes of 10 and tested on the grid size of 20, the success rate is above 98% for 2D and 88% for 3D. As expected, we observe worse performance of the models trained on smaller grids and tested on larger grids since smaller grids do not include examples of longer paths that are possible in larger grids.

Although the network in most of the cases is able to predict a feasible path (which leads from the start- to the end-point), paths are not always optimal. In Figures 4B,E, we observe that we obtain fewer optimal paths when testing on larger grids of size *n* = 20. The red bars in Figures 4B,E show this as compared to smaller grids of size *n* = 10 (blue bars), which produce better results. This is due to the fact that paths in larger environments on average are longer than in smaller environments, and thus more prone to prediction errors. For the same reason, we can also see that the number of optimal paths increases if larger environments are used for training. Thus, results suggest that training on larger maps is more beneficial.

Next we checked how much longer non-optimal paths are with respect to the shortest, optimal path. As in the previous cases, better performance is obtained if trained on the same or larger grids than the test case (Figures 4C,F). Nevertheless, in most of the cases non-optimal paths are on average <10% longer compared to the shortest path (in a range between 5 and 7% for 2D, Figure 4C; and between 7 and 9% for 3D, Figure 4F).

### 4.4. Comparison to Other Algorithms

**Figure 5:**
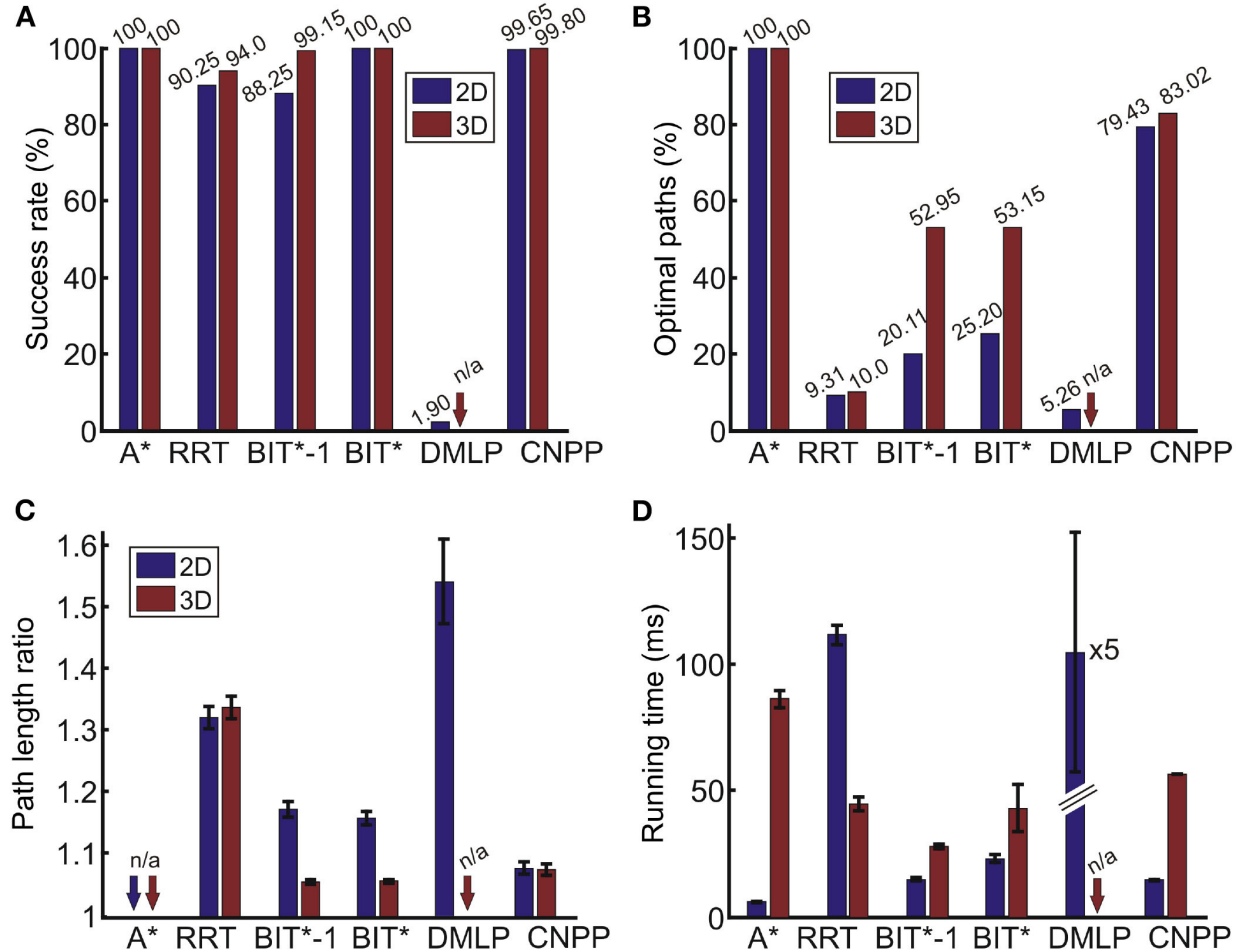
Comparison of different approaches obtained from 2,000 unseen 2D and 3D environments. **(A)** Success rate *SR*, **(B)** percentage of optimal paths *OP*, **(C)** average path length ration of non-optimal paths *LR* (not applicable for the A^*^ since all paths are optimal), and **(D)** average run-time. Error bars denote confidence intervals of the mean (95%). A^*^, A-star algorithm (Hart et al., 1968); RRT, rapidly-exploring random tree algorithm (LaValle, 1998); BIT^*^-1, batch informed trees with a single batch (Gammell et al., 2020); BIT^*^, batch informed trees with multiple batches (Gammell et al., 2020); DMLP, deep multi-Layer perceptron (Qureshi et al., 2019); and CNPP, convolutional network for path planning (our method).

Some comparisons of our method (CNPP) to other approaches for single-path planning are presented in Figure 5. For comparison we used four different algorithms: A^*^ (Hart et al., 1968), Rapidly Expanding Random Trees (RRT, LaValle, 1998), DMLP (Qureshi et al., 2019) and Batch Informed Trees (BIT^*^, Gammell et al., 2020). All these algorithms have been successfully applied to path finding problems. Note that RRT^*^ (that finds optimal paths but is slow) has not been included in this analysis, because BIT^*^ has been shown to be faster and equally optimal (Gammell et al., 2020). The results presented below will show that they perform quite differently (note that we tuned parameters of each algorithm to obtain the best performance). In section 5, we will expand on this, also discussing under which conditions one or the other algorithm might be beneficial.

For the 2D case, we used 30 × 30 grids and, for the 3D case, grids of size 20 × 20 × 20. The CNPP had 31 layers for 2D and 21 layers for 3D.

For the RRT, we limited the search to a maximum of 1,000 samples and a trial was counted as unsuccessful if the goal was not reached within this limit. The other parameters were as follows. The maximum distance between a new node and the nearest node was 2, and the goal bias probability was 0.1.

For BIT^*^, we used 500 and 1,500 samples for the initial batch for 2D and 3D, respectively. The batch size was increased by 500 samples for the next batch for both cases until the path was found. We also performed path search with a single batch (first batch, noted as BIT^*^-1). In this case a trial was counted as unsuccessful if the path was not found using this batch. In all cases, the maximum distance for the node neighborhood was set to 3.

In case of DMLP, we skipped the encoder network (Qureshi et al., 2019) and only used the planning network since we did not use point clouds for obstacle representation. In our case, we used the same obstacle representation (only flattened) as in CNPP with additional current- and end-point coordinates, thus, the total length of the input vector was 904). The DMLP consisted of 14 hidden layers with 1,792, 1,536, 1,408, 1,280, 1,024, 896, 768, 512, 384, 256, 256, 128, 64, and 32 neurons per layer, respectively. For the path planning step, we limited the search to 100 iterations where one iteration consists of predicting the next states from both the start and goal locations and a collision check (see Qureshi et al., 2019 for more details). Also, we counted a trial as unsuccessful if the path length ratio *LR* was more than 1.75. All other parameters were kept as in the original implementation provided by the authors.

Figure 5 shows that the performance of CNPP and BIT^*^ is comparable to A^*^ with respect to the success rate. Note that RRT and BIT^*^ are not parameter-free. For RRT there is no parameter set with which a performance similar to our CNPP can be obtained on such mazes. After some tuning, the best performance, as measured by *SR*, *OP*, and *LR*, is shown in the figure, where paths are not optimal in most of the cases.

BIT^*^ and BIT^*^-1 perform in general better than RRT. BIT^*^ allows reaching 100% success rate, however, the percentage of optimal paths is much lower as compared to CNPP, where BIT^*^ is slower than CNPP in 2D (*t*−*test, p* < 10^−6^) and faster than CNPP in 3D (*p* < 0.01). For BIT^*^-1 we tuned the parameters so that similar (2D, *p*>0.1) or faster (3D, *p* < 10^−6^) run-time performance as compared to CNPP was obtained. Then, however, the percentage of optimal paths for BIT^*^-1 is also much lower than for CNPP. The percentage of optimal paths for BIT^*^-1 and BIT^*^ could be improved by increasing the number of samples in the batches, however, this would increase search time.

With respect to the path length ratio (see Figure 5C), A^*^ always returns optimal paths and outperforms all other algorithms in both 2D and 3D, however, it is slower than all other, except DMLP, algorithms in 3D (*t*−*test, p* < 10^−6^ for all cases). In the 2D case, CNPP returns on average shorter paths than RRT, BIT^*^-1, BIT^*^, and DMLP (*p* < 10^−6^ for all cases), whereas in 3D, paths of CNPP on average are longer than BIT^*^-1 and BIT^*^ paths (*p* < 10^−6^ for both cases), but shorter than RRT paths (*p* < 10^−6^).

The performance of DMLP remains far inferior to all other algorithms. Due to this reason, we considered DMLP only for the 2D case.

Note, however, that measuring computational time has to be taken with a grain of salt, as this might strongly depend on the efficiency of the implementation. To make a fair comparison, all algorithms were implemented in Python and we have always used the same hardware to test run-time of all algorithms. Hence, while absolute values will change with implementation and hardware, relations between the different algorithms should remain similar if not using fundamentally different hardware (like parallel processing, etc.).

When averaging over 2,000 different single paths, we find that under these conditions the A^*^ algorithm is the fastest in 2D (*t*−*test, p* < 10^−6^ when compared to all other algorithms). CNPP and BIT^*^-1 are slower than A^*^ but faster than RRT and DMLP (Figure 5D, *p* < 10^−6^). In 3D, A^*^ is slower than RRT, BIT^*^-1, BIT^*^, and CNPP (*p* < 10^−6^ for all cases) but always optimal. RRT, BIT^*^-1, and BIT^*^ are faster than CNPP (*p* < 10^−6^ for RRT and BIT^*^-1, and *p* < 10^−6^ for BIT^*^) but percentage of optimal paths is much smaller as compared to CNPP (see Figure 5B). For example, in 3D RRT returns many paths that climb up and then the path runs along the “open-air space” on top of the maze (remember that these environments look like city maps with buildings standing on the ground). CNPP, on the other hand, renders in 2D and in 3D quite a good trade off between speed, which is a bit slower, and optimality, which remains quite high. Note that, as we will show below, although CNPP is not the fastest on single path predictions, it outperforms A^*^ and BIT^*^ on multi path predictions (see Figure 7).

Specifically note that on average the run-time of DMLP in 2D is five times slower as compared to the RRT. This is due to the fact that, in case of DMLP, the network has to be exploited many times to plan the complete path.

In summary, A^*^ algorithm gives optimal solutions, but does not scale well when dimensions increase, e.g., 2D vs. 3D case (see Figure 5). For this reason, algorithms such as RRT and BIT^*^ were developed for robotic applications, in particular for trajectory planning for manipulation tasks in 3D environments, which do not provide optimal solutions but are faster than A^*^. Here we showed (see Figure 5) that in some cases we outperform these algorithms with respect to success rate and/or path optimality and/or time.

All these observations also show that for most algorithms there is a speed vs. path length trade-off. Furthermore, parameter tuning can—according to our experience—take quite some time and will have to be re-done whenever the structure of the environment changes. This will be discussed more in the Discussion section arguing that CNPP is less affected by this problem.

### 4.5. Prediction of Multiple Paths

One core contribution of this study is that our approach can be used for multi-path prediction without training on this.

**Figure 6:**
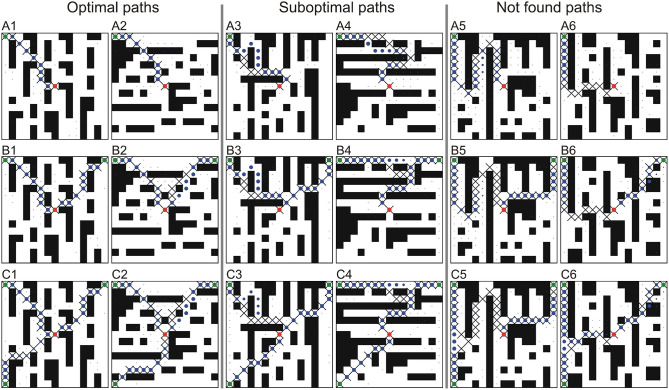
Examples of multi-path predictions on six unseen environments of size 15 × 15. For each environment we show: **(A)** single path predictions (control case), **(B)** predictions of two paths, and **(C)** prediction of three paths. Crosses denote A^*^ solutions where blue dots denote predicted paths using the CNPP. Size of the dots correspond to small (close to zero) and large (close to one) values of the network output. Green and red dots correspond to start-points and end-point, respectively.

Accordingly, examples for the predictions of two and three paths are shown in Figure 6. Note that the first row is a control case. As above, also here we show cases of optimal, sub-optimal and not-found paths. The following four cases can be observed.

1. Paths are not disturbed by adding a second or third source, which usually leads to optimal solutions (see optimal paths in Figures 6A1–C1).
2. Network predictions can change leading to sub-optimal paths, when adding more sources. For example, CNPP may find a single optimal path (Figure 6A2), but then return a sub-optimal path when additional start locations are included in the input (Figure 6B2).
3. A path can “disappear” when adding a second or third source (see Figures 6A4,B4) or, the other way around,
4. a path can “appear” when adding more sources (see Figures 6A6,C6).

A statistical evaluation for the multi-path prediction is given in Table 2, where we show the performance for prediction of one (control case), two and three paths tested on the 15 × 15 grid. Results show that the success rate of finding two paths out of two searched paths and three paths out of three searched paths is 96.4 and 83.9%. Thus, performance is slightly decreasing with increasing number of searched paths. On the other hand, in the case of three-path-search the network was always able to predict at least one path, and success rate for the prediction of two paths was relatively high, i.e., 99.2%. Note that we get an improvement compared to two-path-search since there is a higher chance to predict two paths out of three than two out of two.

**Table 2:** Results for the prediction of multiple paths obtained from 1,000 unseen 2D environments.

	**Number of searched paths**		
	**1**	**2**	**3**
**Success rate (%)**			
1 path found	**99.50**	99.80	100
2 paths found	N/A	**96.40**	99.20
3 paths found	N/A	N/A	**83.90**
**Optimal paths (%)**			
	93.77	85.88	83.33
**Path length ratio (Mean** **±** **CI [95%])**			
	1.07 ± 0.009	1.15 ± 0.023	1.22 ± 0.029

*Error margins denote confidence intervals of the mean. Bold numbers correspond to the cases when searched for one, two and three paths, respectively*.

Regarding path optimality, we observe that we get fewer optimal paths (85.88 and 83.33% for two-path and three-path search, respectively) and that paths become longer with increased number of searched paths (15 and 22% for two-path and three-path search, respectively). This is due to the fact that in the case of multiple sources, the paths are more prone to intersect and this way become less optimal. However, given the fact that the network has never been trained on multiple paths, it shows a surprisingly good performance. Naturally, results should improve by training the network on multiple paths, but the goal of this study was to show how far one can even get by not doing this.

### 4.6. Run-Time Estimates

We compared our algorithm to A^*^ (the fastest algorithm in the 2D case) and BIT^*^ (fastest in 3D). We have chosen BIT^*^ against BIT^*^-1, since it has a 100% success rate and was still faster than CNPP according to the single path-results in Figure 5. For CNPP, we used 21 layers on a 30 × 30 grid in 3D and 31 layers on a 20 × 20 × 20 grid in 2D.

**Figure 7:**
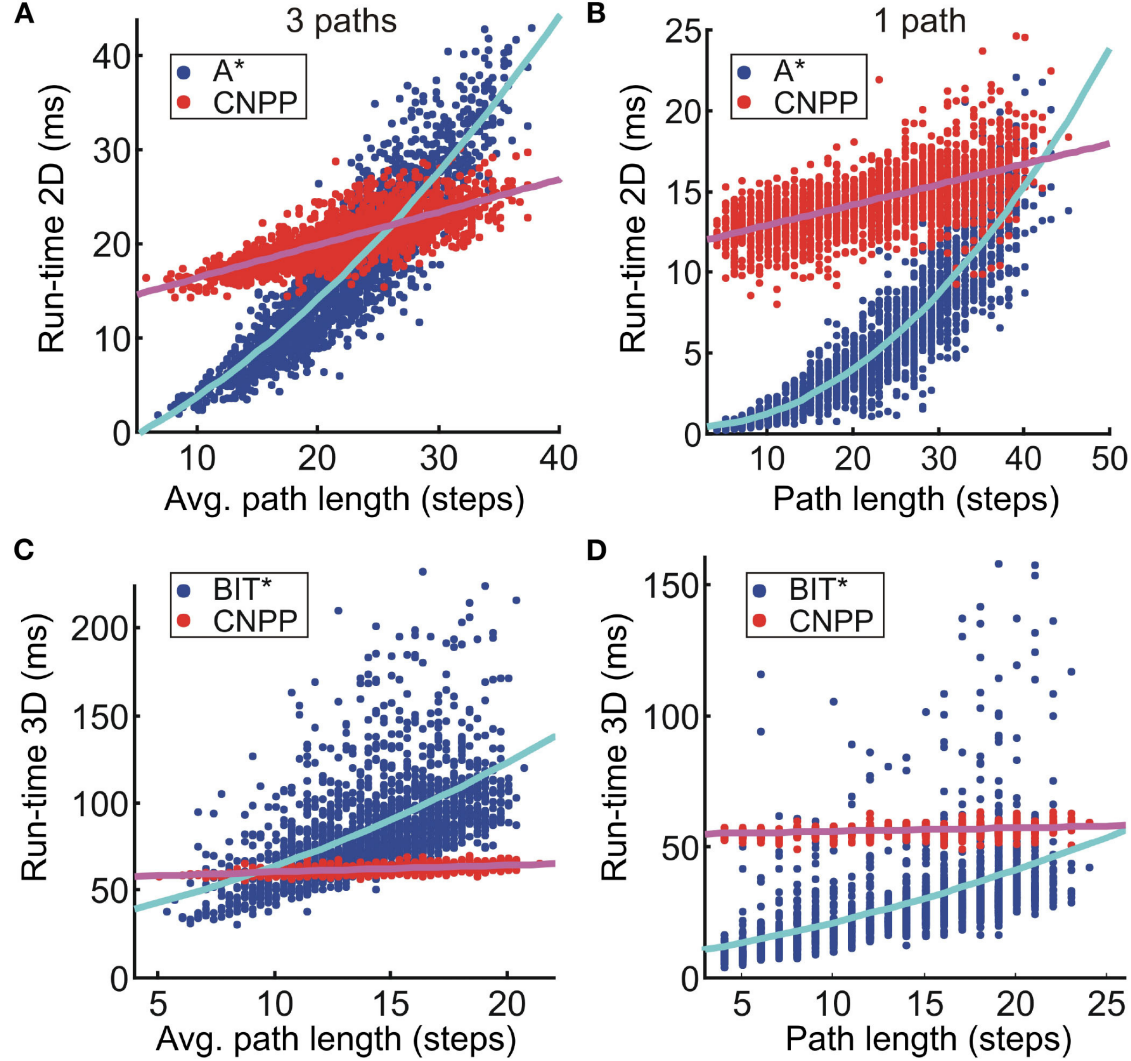
Results for the run-time comparison between **(A,B)** A^*^ and CNPP (2D case), and **(C,D)** BIT^*^ and CNPP (3D case). Data obtained from 2,000 unseen 2D environments of size 30 × 30 and 3D environments of size 20 × 20 × 20. **(A–D)** Show run-time for the search of three paths and one path, respectively. We used a first order and second order polynomial to fit A^*^/BIT^*^ and CNPP data, respectively.

In Figure 7, we show a run-time comparison between A^*^ and CNPP for planning three paths (Figure 7A) and one path (control case, B) in 2D, whereas a comparison between BIT^*^ and CNPP in 3D is shown in Figures 7C,D. We show the run-time of each realization (in total 2,000) for all cases. Note that the total run-time consists of search/prediction time (*t*_*search*_/*t*_*pred*_) plus path reconstruction time (*t*_*rec*_). For A^*^ and BIT^*^, the search time is quadratic and the path reconstruction time is linear with respect to the searched path length, whereas for the CNPP, prediction time is constant and path reconstruction time is linear. As expected, we see that on average the total run-time of A^*^ and BIT^*^ increases quadratic if paths are getting longer. The total run-time of CNPP in 2D on average increases linearly due to increase of the reconstruction time. In 3D, this linear increase can be neglected, because it is very small relative to the prediction time. Results for 4-path prediction (Figures 7A,C) show that in 2D on average CNPP is faster than A^*^ if paths are longer than 26 steps (see Figure 7A), and, in 3D, CNPP is faster than BIT^*^ already for short paths (longer than 9 steps, see Figure 7C).

Note that for *k* paths, the total time for A^*^/BIT^*^ is *k* × (*t*_*search*_ + *t*_*rec*_), whereas for the CNPP it is *t*_*pred*_ + *k* × *t*_*rec*_. Reconstruction time is much shorter than search/prediction time and, thus, this makes CNPP faster compared to A^*^/BIT^*^ when the dimension of the grid or the number of the searched paths increases.

As already shown in Table 1, networks with more layers are needed to process larger grids. Results suggest, that the number of required layers approximately corresponds to the linear grid size, e.g., for a 10 × 10 environment we need approx. 10 layers, etc. Thus, assuming a grid with *n* × *n* nodes, numbers of layers *n*, number of filters *f*, and also assuming that all neurons in one layer can be processed in parallel on the GPU in time *n*^2^/*p* with *p* processing units, this leads to an expected runtime $O\left(n,p,f\right)=\frac{n^{2}}{p}fn$.

### 4.7. Application to City Maps

**Figure 8:**
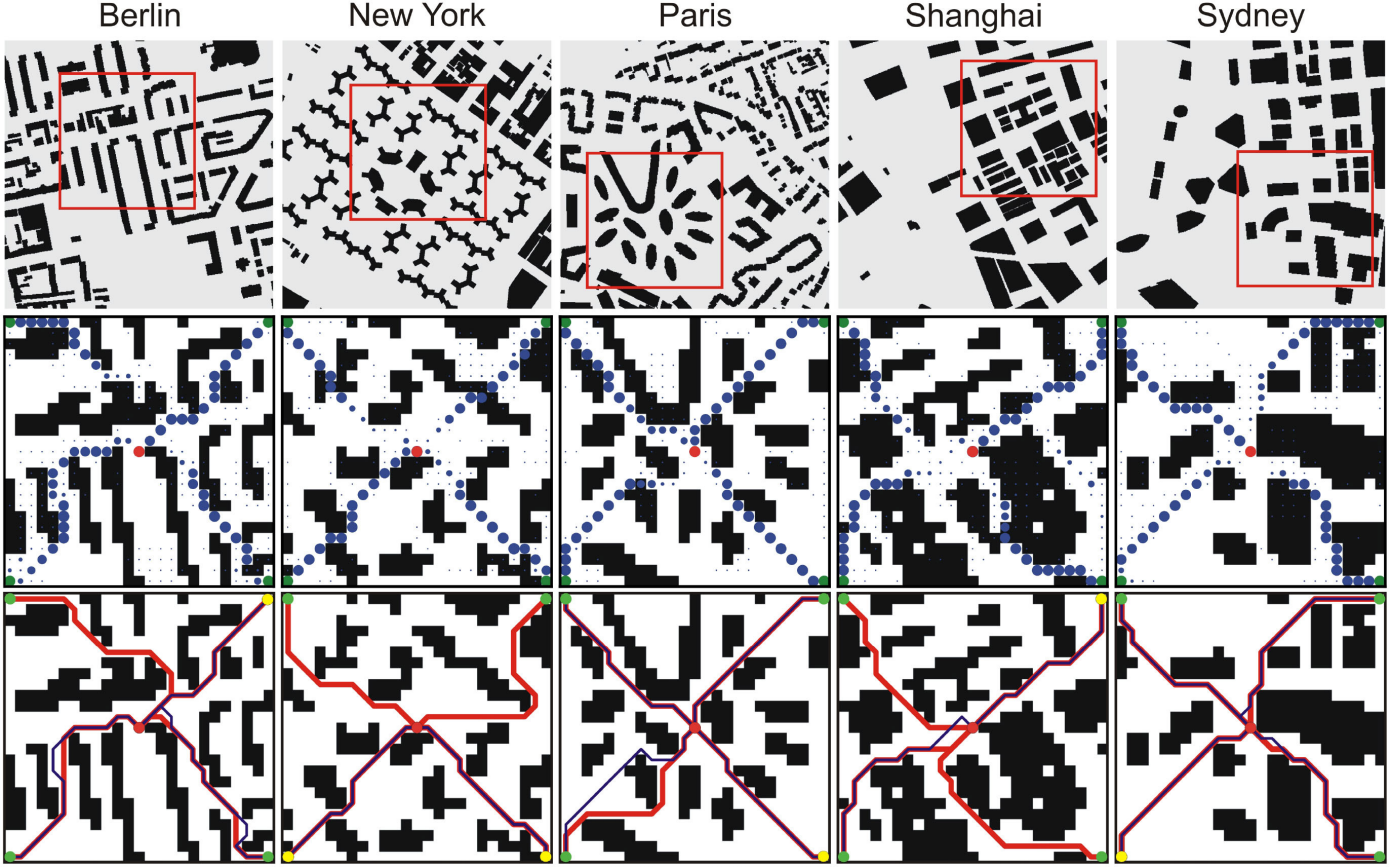
Results for the prediction of four paths in five cities. **(Top)** Original binary city maps, 256 × 256 pixels (Sturtevant, 2012). Selected areas, marked with red rectangles, were down-sampled to 25 × 25 pixels and used for testing on the 2D network trained on 20 × 20 grids. **(Middle)** Network outputs. Blue dots denote predicted paths where green and red dots correspond to start-points and end-point, respectively. **(Bottom)** Decoded paths from the network output (blue trajectories) and A^*^ solutions (red trajectories). Yellow dots denote start-points with shortest paths to the goal.

Results of our approach used on real city maps are shown in Figure 8. We would like to stress that in this case the network was only trained on our synthetic environments of size 20 × 20 that look structurally quite different as compared to these city maps. We can see that only two feasible paths were found in the New York and Shanghai city map, three feasible paths were found for Berlin, and all four paths were found for Paris and Sydney. However, in all cases, the found paths were also shortest paths. These results demonstrate that transfer and generalization from synthetic to city map data is also possible.

## 5. Discussion

In this work, we have presented a novel approach for the generation of single as well as multiple paths. To the best of our knowledge, this is the first approach that allows planning complete multiple paths, while running the network's prediction only once, which is reminiscent to perceptual pop-out phenomena in humans. Note that most of the afore discussed deep-learning approaches generate paths iteratively and only deal with planning of single paths (Tai et al., 2017; Panov et al., 2018; Bency et al., 2019; Qureshi et al., 2019) or they generate multiple-paths for each agent separately (Chen et al., 2017; Long et al., 2017; Everett et al., 2018) but only deal with collision avoidance path planning and not with path planning for navigation in maze-like environments.

Recently, Pérez-Higueras et al. (2018) proposed an approach, which is also able to plan paths in one-shot using a fully convolutional network, however, they only deal with single path planning for human-aware collision-free navigation in simple environments. Also, they use a two-step approach where first the network is used to predict the path and then RRT^*^ (Karaman and Frazzoli, 2011) is used on top of it to refine the predicted path, which is computationally more expensive than the here used simple path reconstruction algorithm (bidirectional search).

Possibly the most interesting finding of our study is that the CNPP method is able to find multiple paths without being trained on them. This could be also used to solve single-source-multi-target (or vice versa) problems. However, we believe that performance could be further improved by indeed including examples of multiple paths in the training set. The other option would be to repeat the network's prediction one or several more times by giving locations, at which the path reconstruction was lost, as new start- and end-points. Moreover, we have also shown that our proposed network can be trained on one size and then used on a different size if the grid sizes do not differ too much and that transfer to city maps is also possible in spite of having used only synthetic training data.

When comparing to other methods, we have observed that CNPP can compete against A^*^ and supersede it in 2D for multi-path prediction as soon as paths get longer. RRT and DMLP perform well in less-cluttered environments (Knispel and Matousek, 2013; Bency et al., 2019; Qureshi et al., 2019), but given mazes, like the ones used here, the CNPP approach clearly outperforms RRT and DMLP. BIT^*^ performs in general better. As mentioned above, BIT^*^ outperforms RRT^*^, which—on these grounds—had not been included in our comparisons (Gammell et al., 2020).

In general, however, such comparisons have to be considered rather carefully, because—as stated above—any given algorithm may be advantageous in certain situations (environments), while performing badly in others. The same is true for speed-estimates, which much depend on the hardware used anyhow.

Another aspect, which has to be considered, is the number of parameters. A^*^ is a parameter free method and, thus, no parameter tuning is required. RRT has three parameters (maximum number of samples, node neighborhood distance and probability of sampling the goal used to make it goal-directed and, thus, faster), and BIT^*^ has also three parameters (number of samples in the initial batch, increment of the samples in the next batches, and node neighborhood distance), which need to be tuned and will influence the performance of the algorithm. Note that BIT^*^ shows asymptotic convergence to the optimal solution. Thus, it converges to the optimal solution as the number of sample increases at the cost of an increasing run-time. RRT, on the other hand, converges almost always to a non-optimal solution. DMLP has three parameters, i.e, number of layers and number of neurons per layer, and dropout probability in the hidden layers during on-line path prediction to introduce stochasticity. Moreover all parametric methods, discussed above, have additional parameters related to the check for a collision-free path between path nodes. In general, this procedure, can be (and usually is) very costly. In comparison, CNPP has three parameters, i.e., number of layers (of which we found that it should correspond to the grid size), and kernel size and number of kernels (suggested to be the same for different grid sizes). Therefore, CNPP is not strongly affected by tuning problems.

The current conclusion, partly arising from results found in the literature, is that tree-based methods may be beneficial in environments with little clutter, for example when doing path planning for robot manipulation. This is in particular valid, whenever path-optimality is not an issue. Different from this CNPP, A^*^ (and similar algorithms) appear the better choice when addressing mazes and as soon as (near) optimal paths are required and little or no parameter tuning is desired.

In our study we have only dealt with static environments and assumed that the complete map of the environment is available beforehand. However, our approach can also be used for on-line planning/replanning in dynamic environments and/or environments where only part of the complete environment is available (perceivable) at any one point in time. In this case, the current position of the robot can be used as the start-point and the next sub-goal (or the final goal, if visible) as the end-point. Thus, path planning toward the goal would be made only for the next few steps, i.e., as far as environment can be perceived or as long as it has not changed.

We believe that useful features of our proposed method such as single-shot multi-path planning, relatively high success and optimality rates, constant run-time, and the ability to generalize to environments of different sizes and types, makes this method an attractive and valuable approach for many applications in robotics as well as other fields.

## Data Availability Statement

The datasets presented in this study can be found in online repositories. The names of the repository/repositories and accession number(s) can be found at: https://alexandria.physik3.uni-goettingen.de/cns-group/datasets/path_planning/.

## Author Contributions

TK contributed to the conception and design of the work, development and implementation of the algorithm, data acquisition, analysis and interpretation of the data, and writing of the paper. SH contributed to the conception of the work, the implementation of the algorithm, data acquisition, analysis and interpretation of the data. TL contributed to the implementation of the algorithm. MT contributed to the analysis and interpretation of the data, and writing of the paper. FW contributed to the conception and design of the work, analysis and interpretation of the data, and writing of the paper. All authors contributed to the article and approved the submitted version.

## Conflict of Interest

The authors declare that the research was conducted in the absence of any commercial or financial relationships that could be construed as a potential conflict of interest.

## Footnotes

^1^Note that path planning consists of two steps, i.e., path search and path reconstruction.

^2^A preliminary version of this paper has been presented at ICRA 2020 (Kulvicius et al., 2020).

^3^The source code will be available online after publication of this manuscript.

^4^More examples of 2D and 3D path predictions can be found at https://alexandria.physik3.uni-goettingen.de/cns-group/datasets/path_planning/.
